# Supplementary material for: Cryo-EM structure of bixafen-bound S. cerevisiae complex II unravels SDHI specificity against pathogenic fungi
Source: Commun Biol. 2026 Jan 28;9:517. doi: 10.1038/s42003-026-09617-8 (PMC13066397; doi:10.1038/s42003-026-09617-8)
Supplement: Supplementary file 1 — Supplementary information [file 42003_2026_9617_MOESM1_ESM.pdf]

Supplementary Information

Cryo-EM structure of bixafen-bound *S. cerevisiae* complex II unravels SDHI specificity against pathogenic fungi

Nikos Pinotsis[1,2*], Claudia Burn-Leefe[1], Sarah Jones[2], Shu Chen[2], Natalya Lukoyanova[2,3], Brigitte Meunier[4], Edward A. Berry[5], Amandine Maréchal[1,2*]

[1] Department of Structural and Molecular Biology, University College London, London, United Kingdom

[2] Institute of Structural and Molecular Biology, Birkbeck College, London, United Kingdom

[3] present address: Structural Biology Science Technology Platform, Francis Crick Institute, London, United Kingdom

[4] Université Paris-Saclay, CEA, CNRS, Institute for Integrative Biology of the Cell (I2BC), Gif-sur-Yvette, France

[5] Biochemistry and Molecular Biology, SUNY Upstate Medical University, Syracuse, NY, USA

* Corresponding authors: a.marechal@ucl.ac.uk; n.pinotsis@ucl.ac.uk

Supplementary Figures 1-15

Supplementary Tables 1-3

Supplementary Text

Supplementary Reference

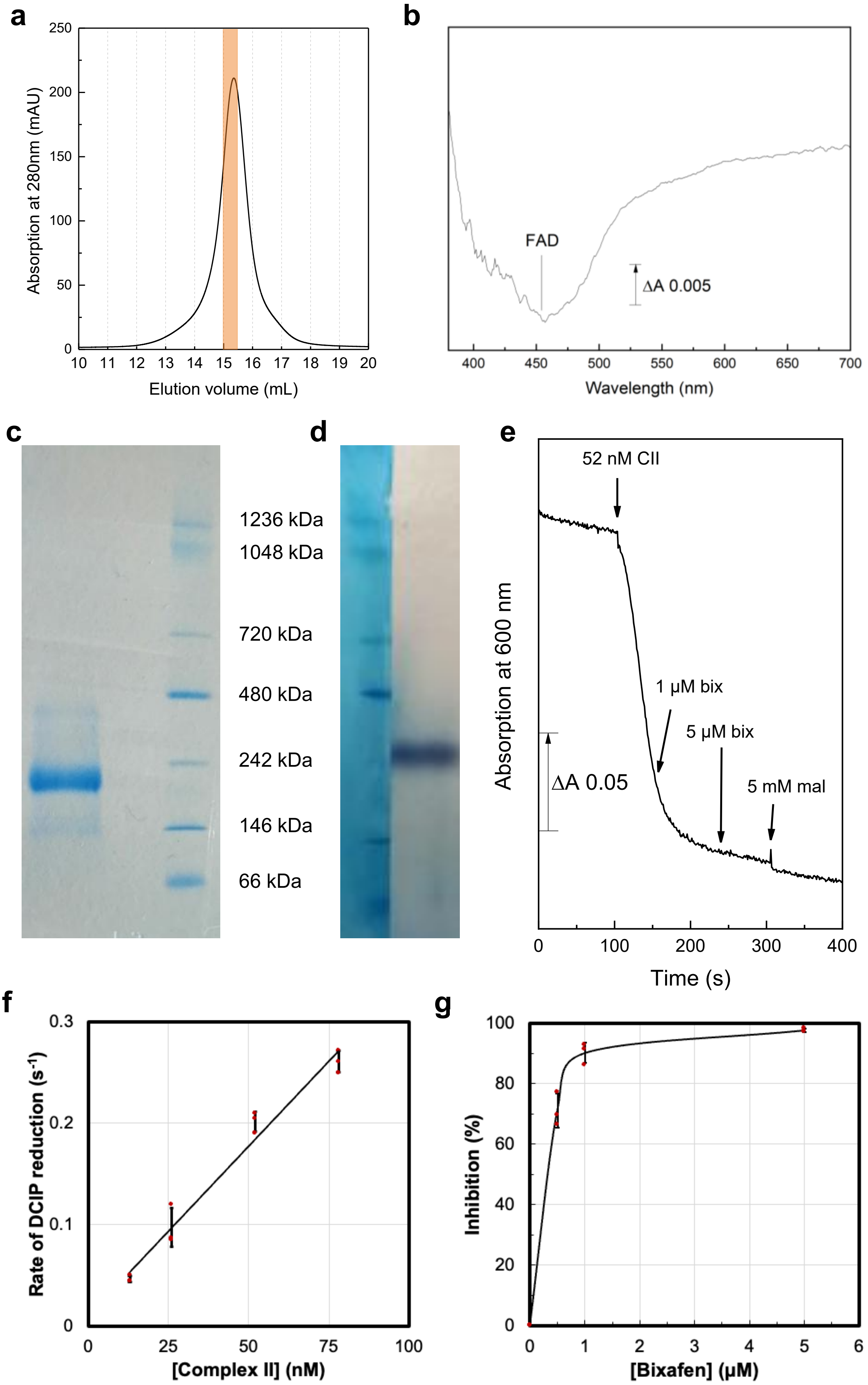

Supplementary Fig. 1: Characterization of the purified CII preparation used for cryo-EM structure determination. a, Final gel filtration elution profile; the peak

fraction (orange) represents the pure CII preparation used for cryo-grid preparation and all subsequently presented biochemical characterization. **b,** Quantification of the FAD content from the reduced-minus-oxidized visible absorption spectrum. **c,** BN-PAGE and **d,** CII in-gel activity performed on a CN-PAGE gel. **e,** Representative trace of a CII activity assay performed following DCIP reduction from the decay of its specific absorption at 600 nm by visible absorption spectroscopy; effect of addition of bixafen (bix) before the assay is stopped by addition of malonate (mal) which targets the CII succinate binding site. **f,** DCIP reduction rate as a function of CII concentration. **g,** CII inhibition rate (in %) relative to mal-inhibited rate as a function of bixafen concentration. Data presented in **f** and **g** are technical repeats performed in triplicates (n=3, red) for each condition; means and standard deviation are displayed in black.

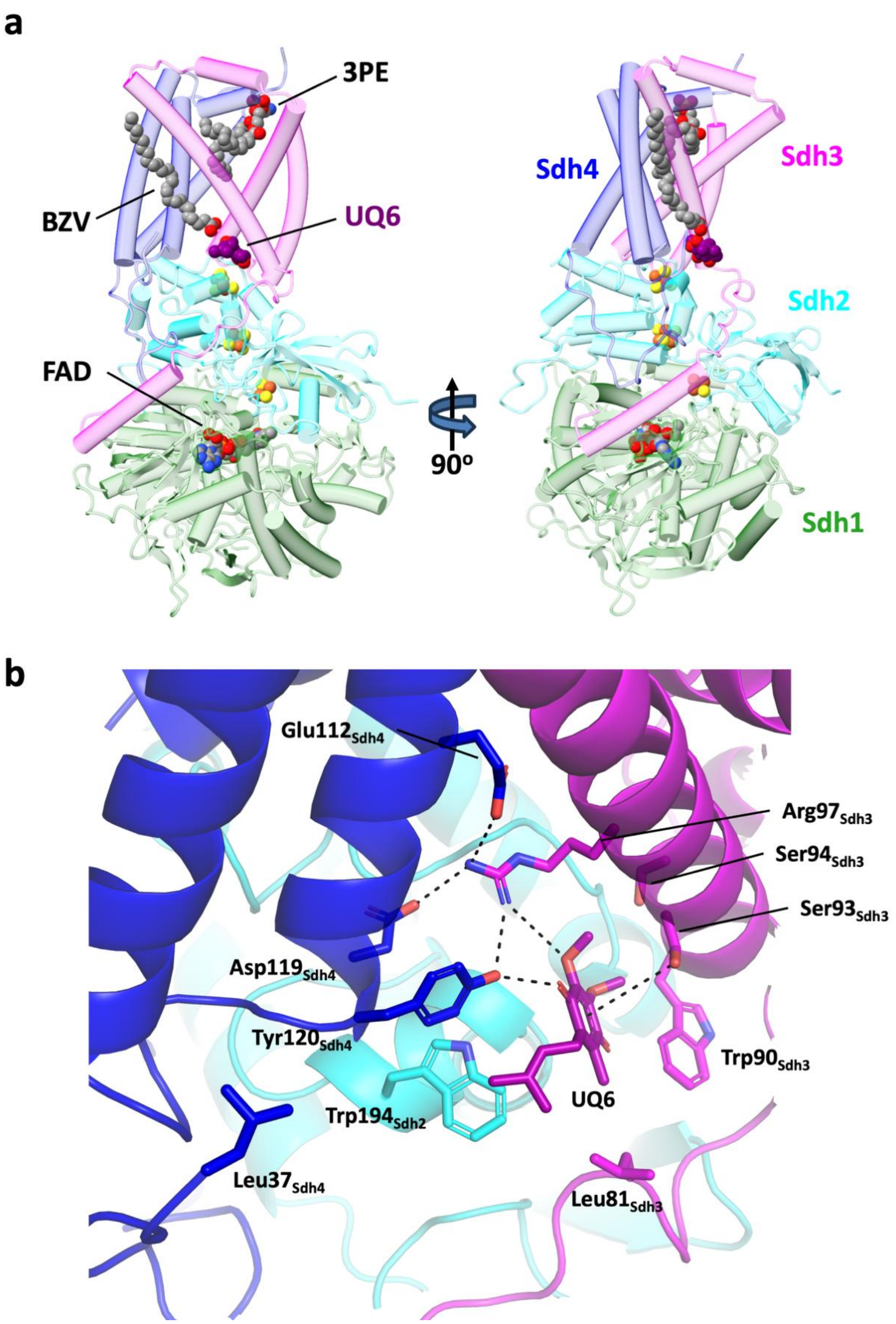

Supplementary Fig. 2: Structure of the *S. cerevisiae* CII with the Q site ubiquinone-6 (UQ6) bound. **a,** Two views of the complex rotated by 90 degrees are shown. Sdh1, Sdh2, Sdh3, Sdh4 and UQ6 are colored in green, cyan, magenta, dark blue and purple, respectively. The lipids 1,2-Distearoyl-sn-glycero-3-phosphoethanolamine (3PE), tetracosanoic acid (BZV), the cofactor (FAD), the Fe-S clusters and UQ6 are shown as spheres. **b,** Cartoon representation of the Q site showing the major interactions of UQ6. Color codes as in panel a.

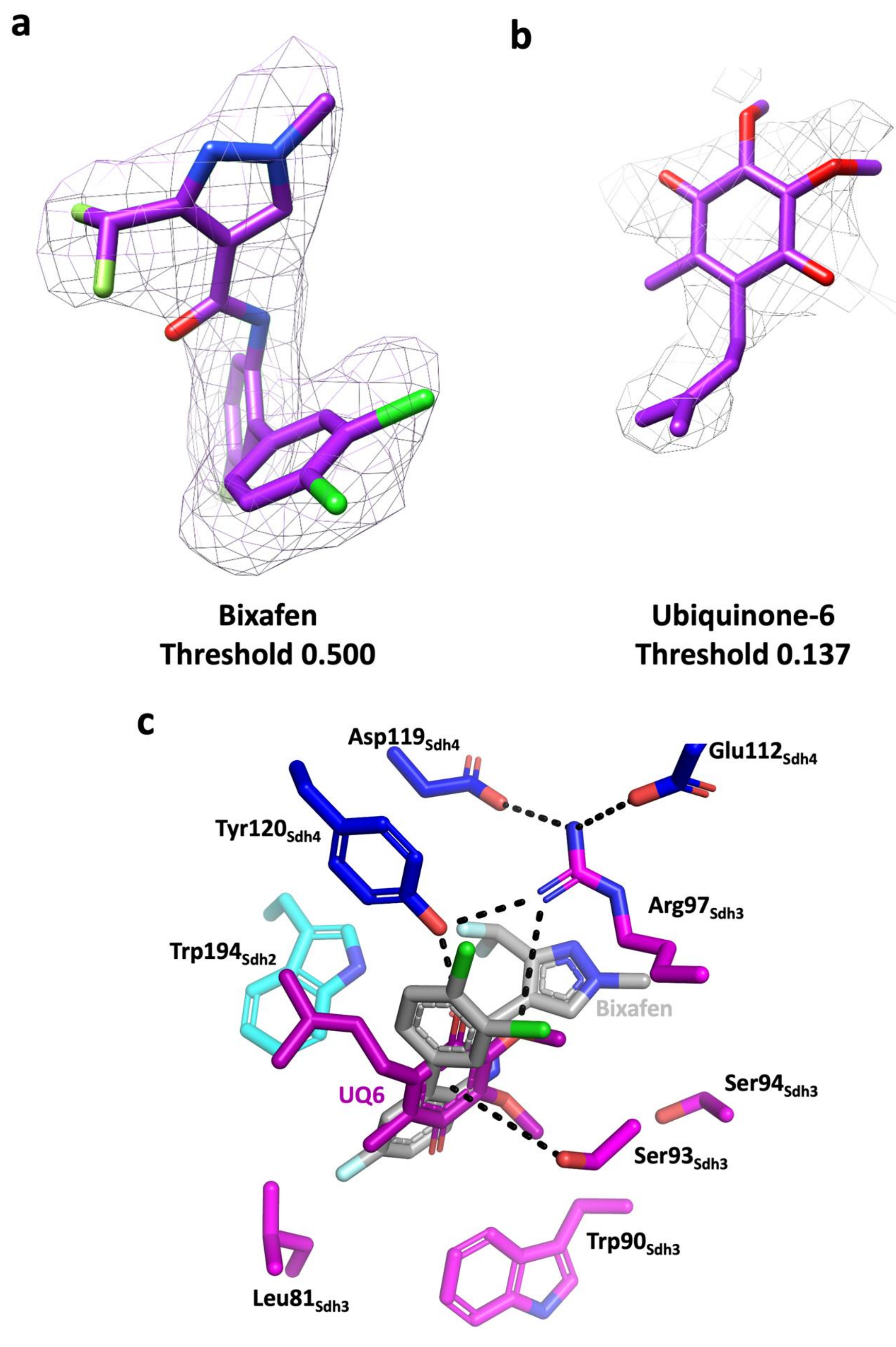

Supplementary Fig. 3: Bixafen and endogenous ubiquinone-6 binding at the Q site of *S. cerevisiae* CII. Coulomb potential density maps of **a,** the inhibitor bixafen and **b,** UQ6 at the Q site. **c**, Overlay of bixafen as seen in the CII-bix structure on the CII-nat structure with bound UQ6. Hydrogen bonds and other electrostatic interactions with UQ6 are shown with dashed lines.

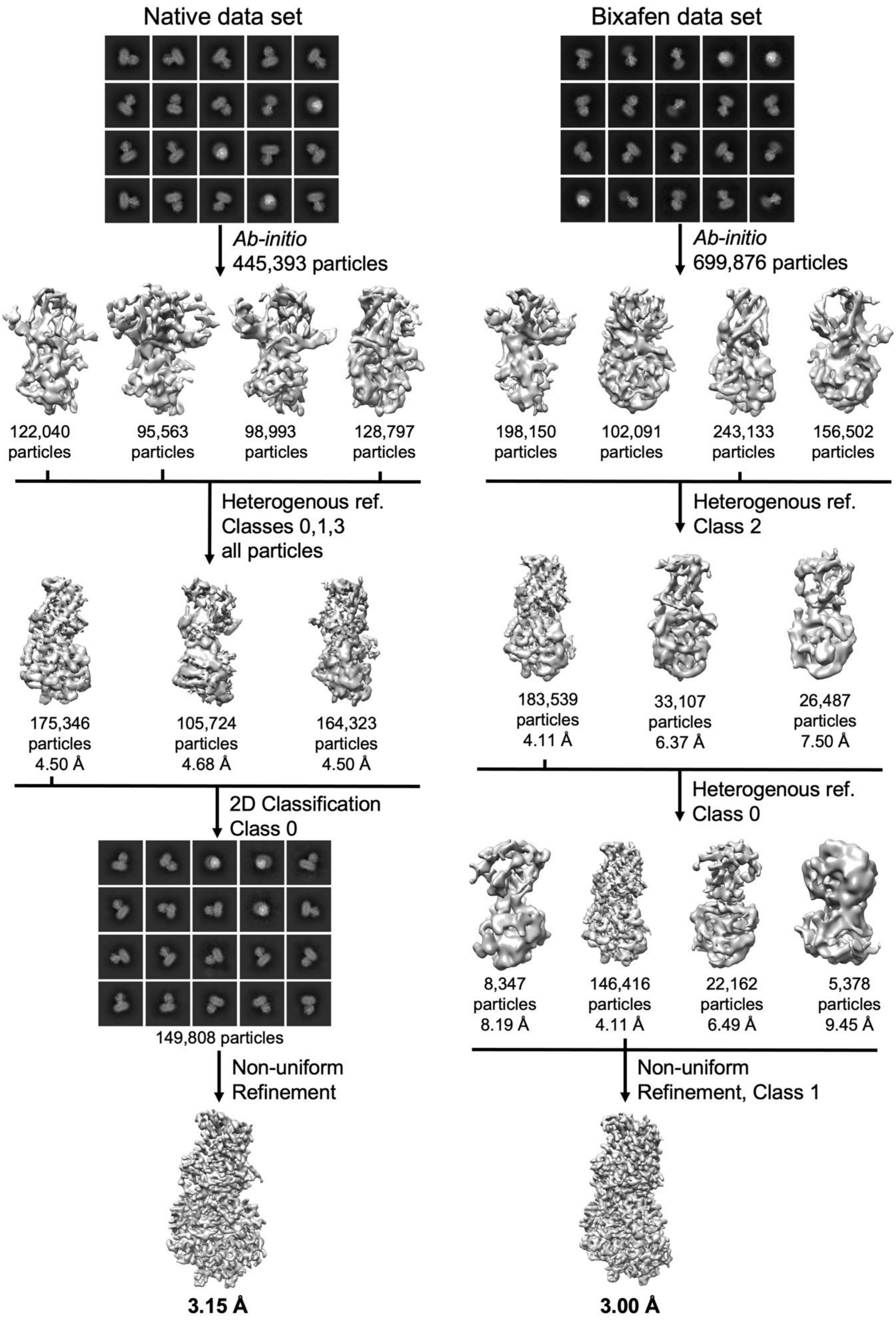

Supplementary Fig. 4: Cryo-EM image processing workflow in cryoSPARC for the CII-nat (left) and CII-bix (right) datasets.

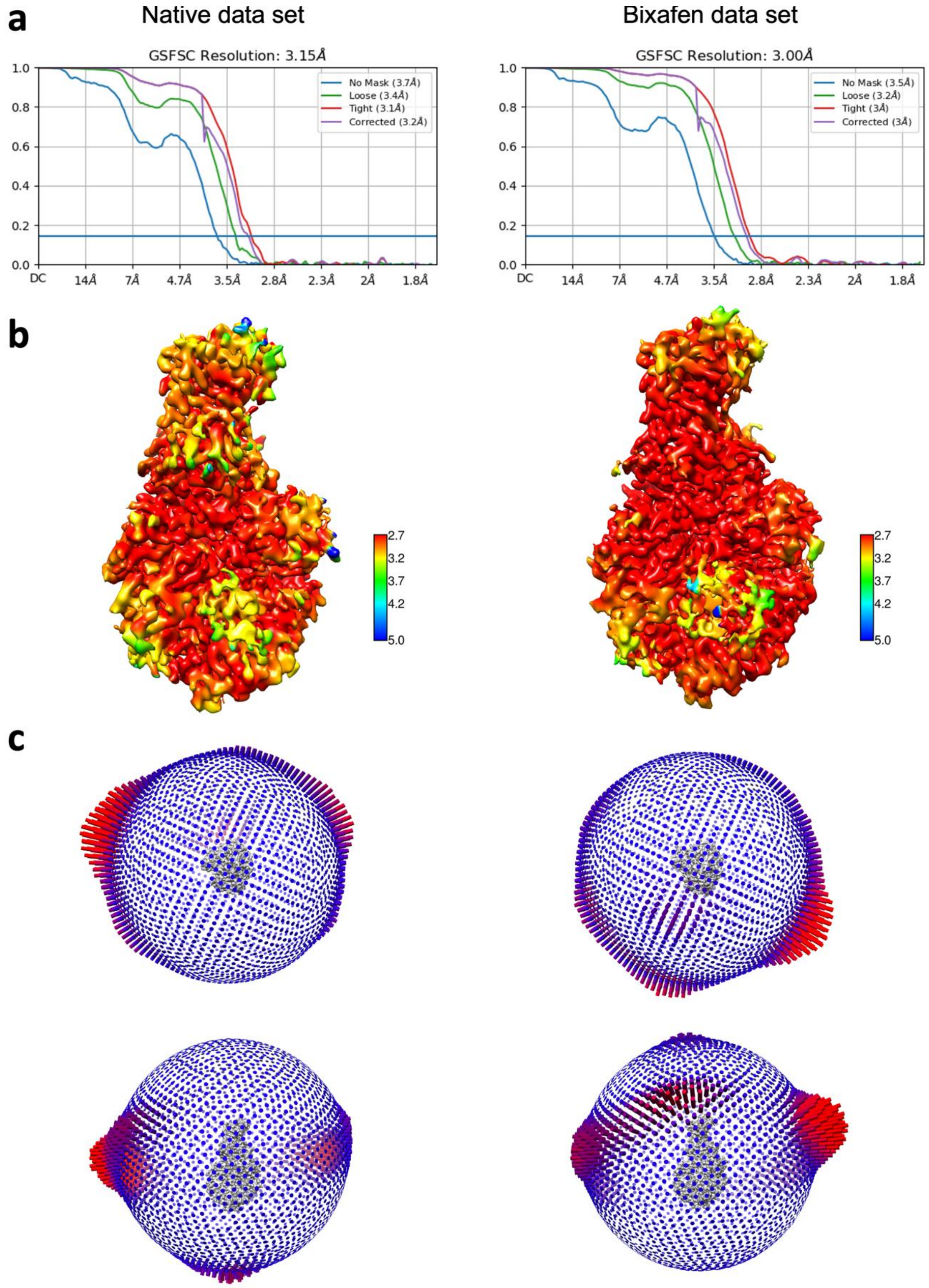

Supplementary Fig. 5: Cryo-EM data validation for the final CII-nat (left) and CII-bix (right) maps. a, Fourier shell coefficient (FSC) plots using different masks. **b,** Local resolution surface rendering maps. **c,** Angulate distribution plots that contributed to the final maps. The plots have the same orientation with each map (upper plots complex shown from its IMS side, lower plots top IMS, bottom matrix). Height and color (from blue to red) are proportional to the number of particles in those views.

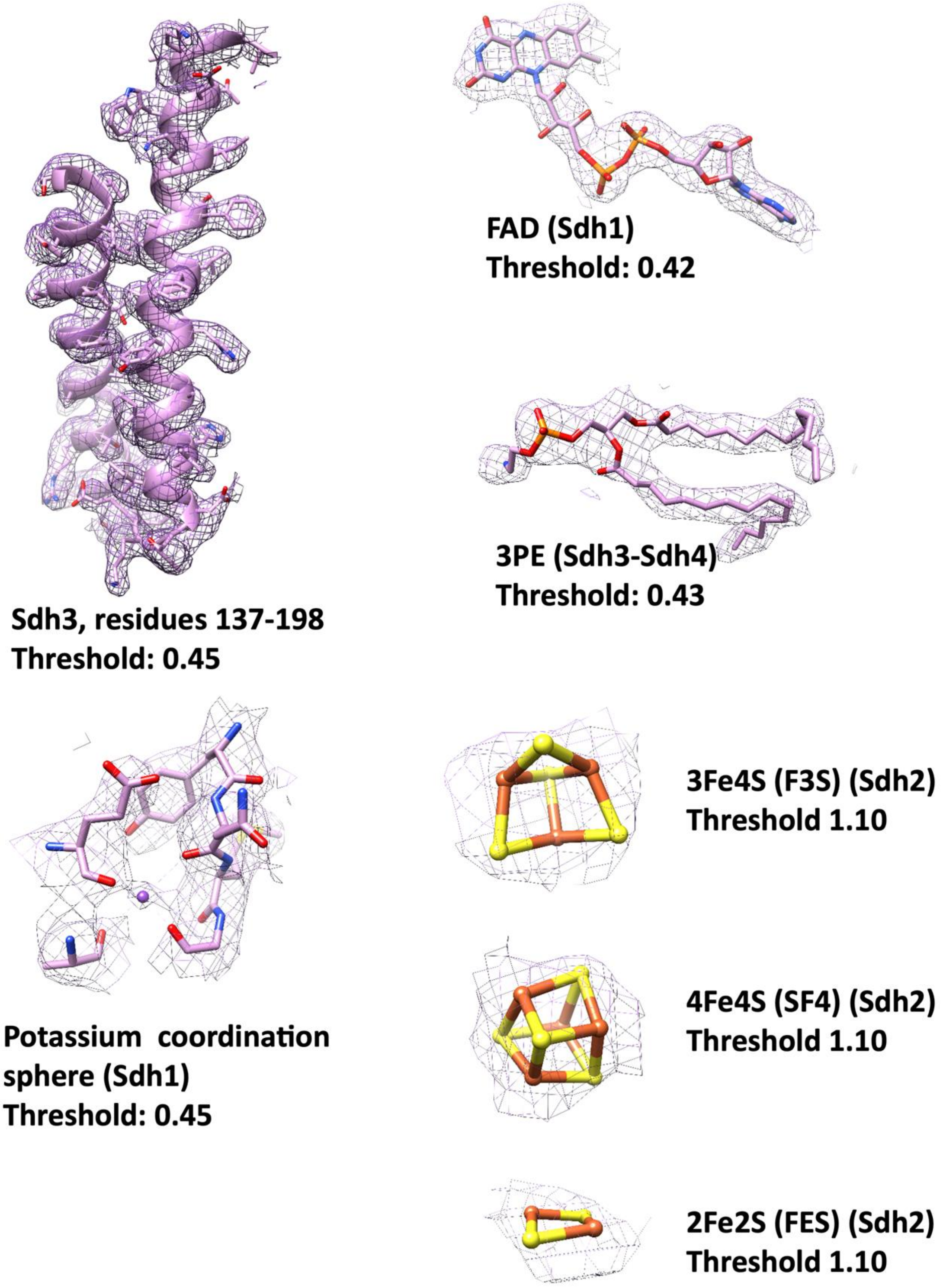

Supplementary Fig. 6: Examples of the map quality for representative key features of CII. Density is shown for an alpha-helix in the membrane domain (Sdh3), the FAD co-factor and potassium ion in Sdh1, the iron-sulfur clusters in Sdh2 and the bound lipid in the transmembrane region of the complex.

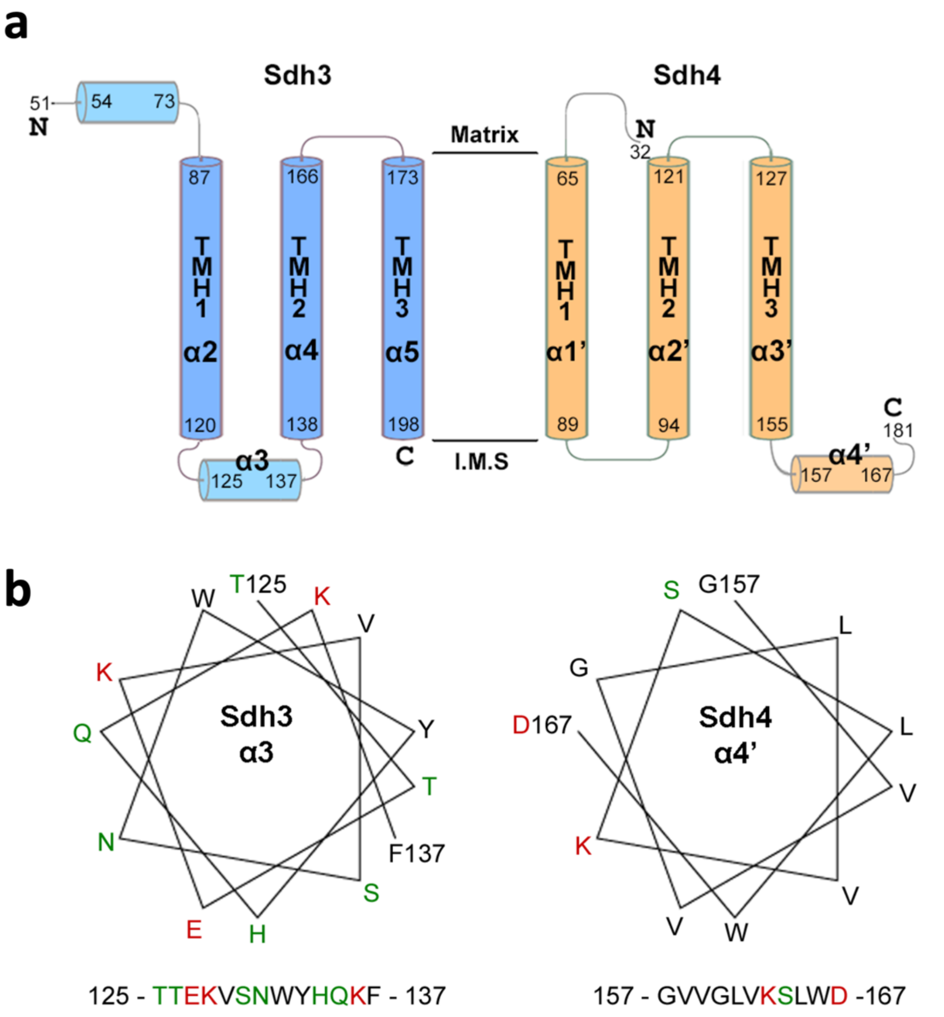

Supplementary Fig. 7: Analysis of CII membrane subunits Sdh3 and Sdh4. a, Topology diagram of the small subunits of *S. cerevisiae* CII. Helices are labeled α1-α5 (Sdh3) or α1'- α4' (Sdh4), and the transmembrane helices are labeled TMH1 - TMH3 for each. Residues numbers at the start and end of each helix are indicated. **b,** Helical-wheel plots showing moderate amphipathy of the in-plane IMS-surface helices.

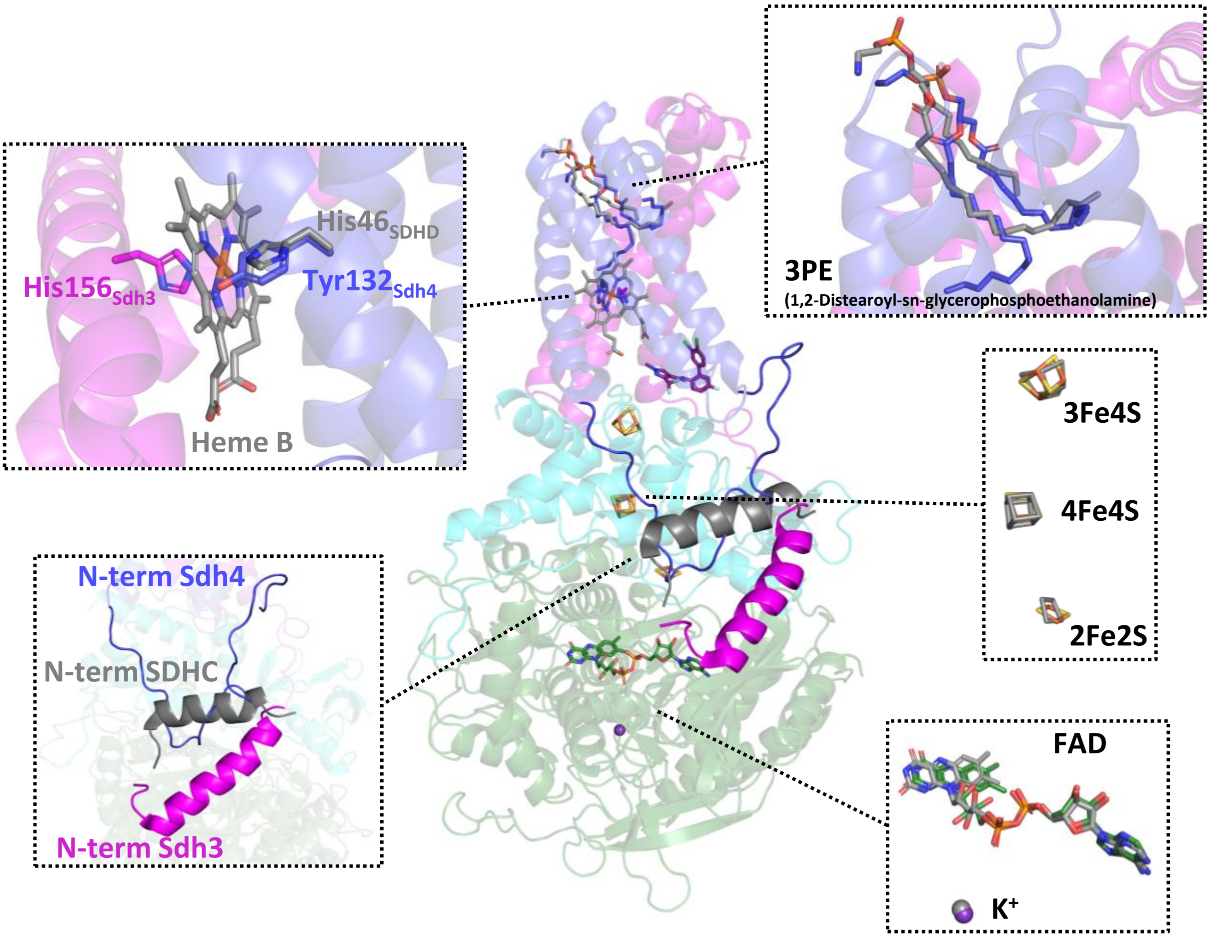

Supplementary Fig. 8: Structural comparison between the yeast *S. cerevisiae* (PDB ID 9QDM) and the avian (PDB ID 6MYO) vertebrate CII structures. The yeast CII structure is coloured as in Fig. 1 while features of the overlaid avian CII structure are highlighted with grey. Major differences are shown in panels to the left and similarities to the right.

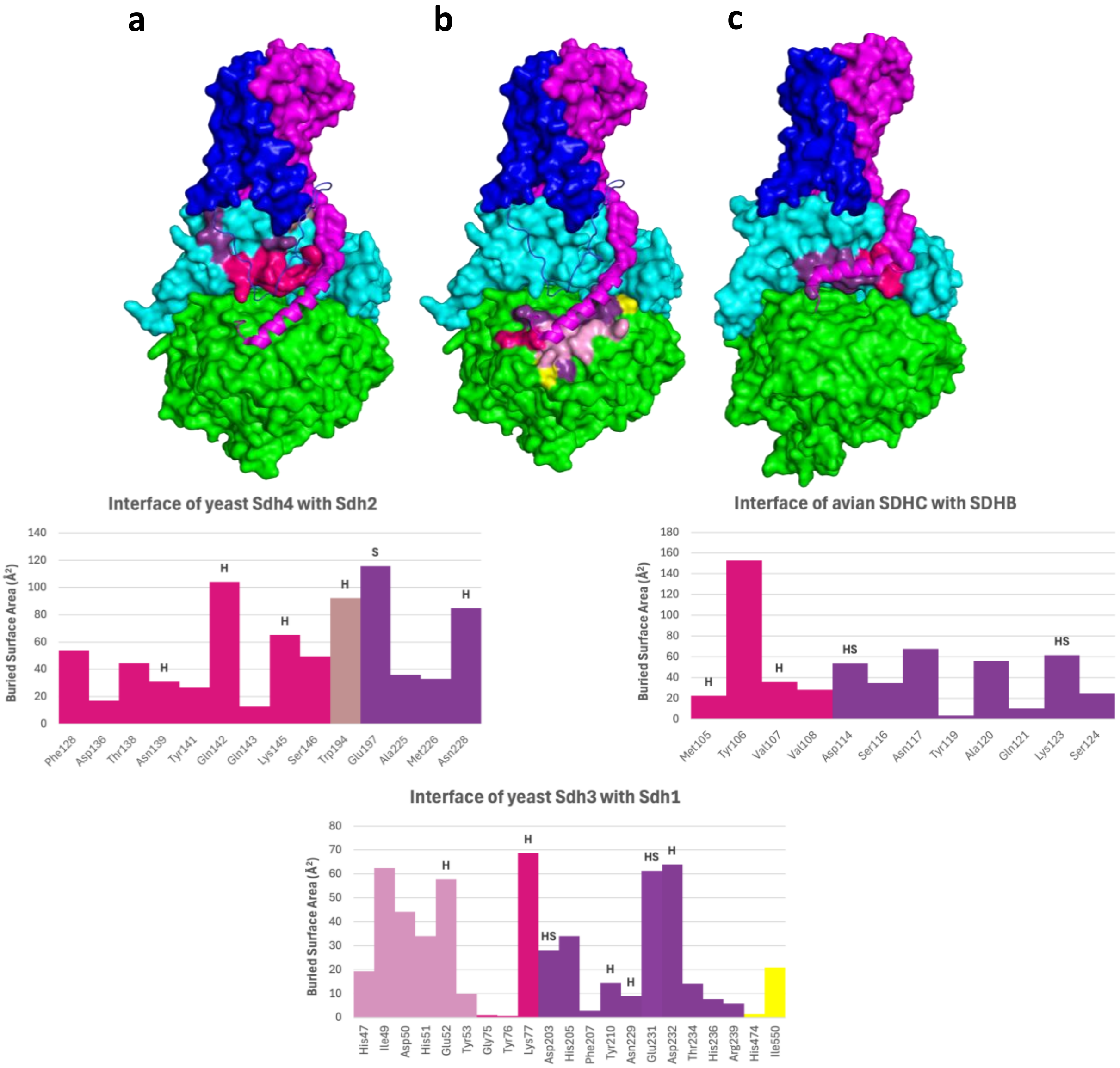

Supplementary Fig. 9: Interaction interfaces of CII hydrophilic subunits with the N-termini of the hydrophobic subunits in the yeast (PDB ID 9QDM) and avian (PDB ID 6MYO) structures. (**a**) Interaction of yeast Sdh4 N-terminal coil with Sdh2 (**b**) Interaction of yeast Sdh3 N-terminal α-helix with Sdh1 (**c**) Interaction of avian SDHC N-terminal α-helix with SDHB. All subunits are represented as surfaces. Sdh1/SDHA are coloured in green, Sdh2/SDHB in cyan, Sdh3/SDHC in magenta, and Sdh4/SDHD in blue. The N-terminal matrix extensions of the hydrophilic subunits are shown as cartoon representations. The interacting surfaces are evidenced with different colours depending on their position on the interacting hydrophilic subunit. The tables below each structure show the buried surface area with each interacting amino acid colour coded as in the structures. Hydrogen bonds and salt bridges are indicated by **H** and **S** above the interacting residues. These may be involved in multiple interactions.

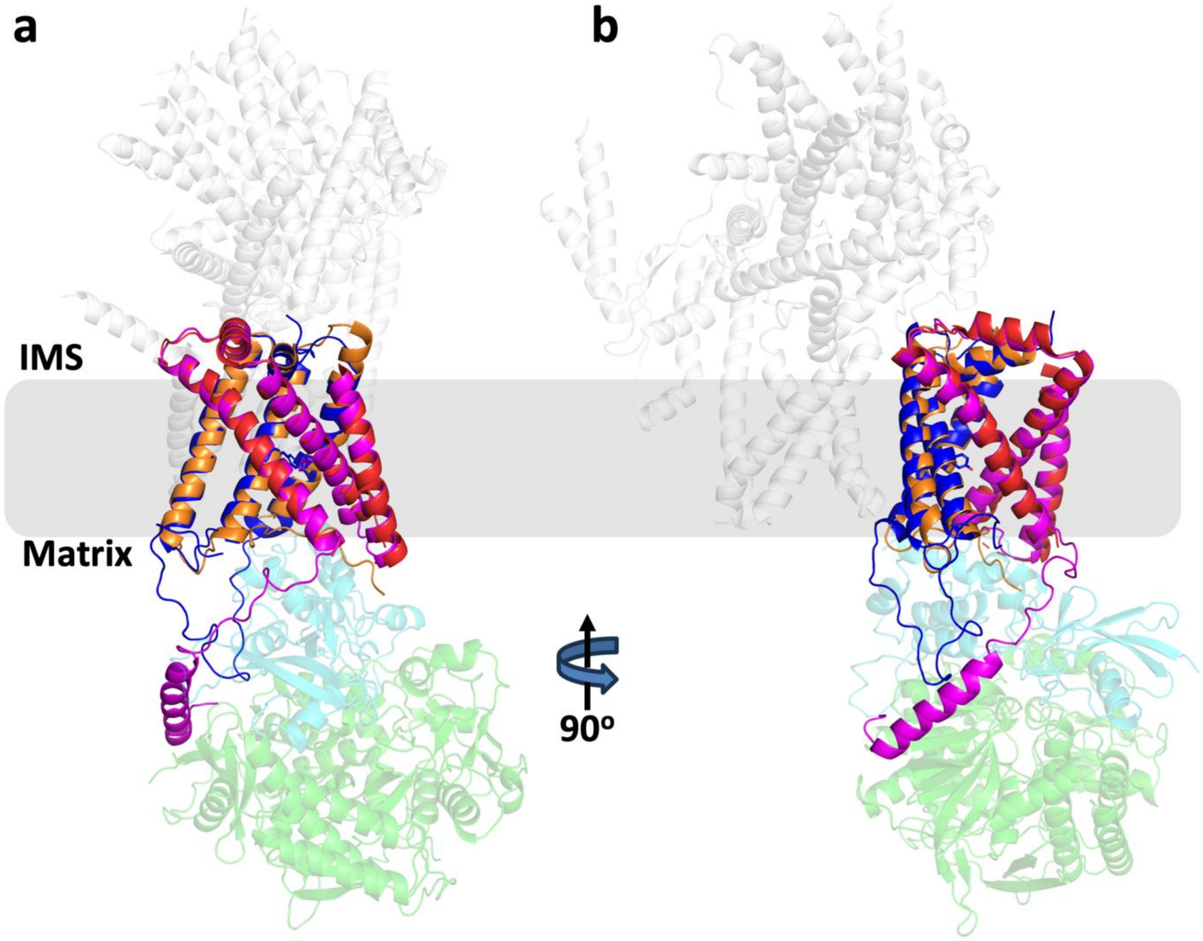

Supplementary Fig. 10: Superimposition of CII with the TIM22 translocation complex. Two views rotated by 90 degrees are shown (**a** and **b**). CII is colored as in Fig. 1, with Sdh3 in magenta and Sdh4 in blue and the Sdh3 and Tim18 from TIM22 are in red and orange respectively. The soluble domains of CII and TIM22 are semitransparent, and they are spanning to the Matrix (CII) and to the IMS (TIM22).

a

```
                   ---------SDH2-------- -------SDH3------ ---SDH4--

                   193        204 234   241 81                98 113  120
                    |           | |       | |                  | |      |
Saccharomyces      WWNQEQYLGPAV YRCHTIMN LTIYQPQLTWYLSSLHRI FNSCITDY
Gallus gallus      WWNGDKYLGPAV YRCHTIMN ISIYKWSLPMAMSITHRG LGQVITDY
                   ***  ::****** ******** ::**: .*.  :*  **  :..  ****
Prim.cons.         WWN222YLGPAV YRCHTIMN 22IY222L2222S22HR2 2222ITDY
```

b

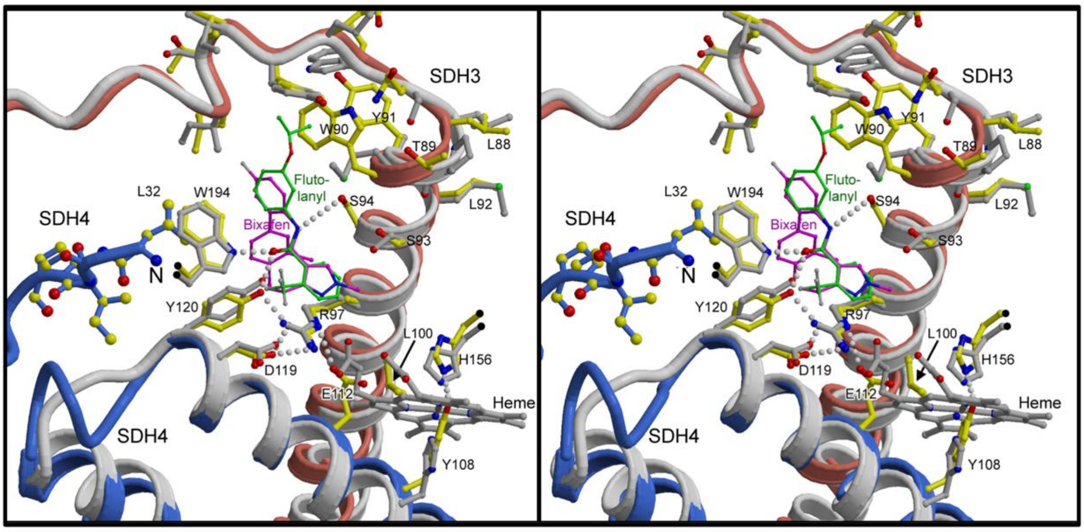

Supplementary Fig. 11. Comparison of bixafen binding in yeast CII-bix with flutolanil binding in the avian structure. a, Sequence alignment of two segments of Sdh2 and one each of Sdh3 and Sdh4 that comprise the surroundings of the inhibitor binding site. The avian structure (PDB ID 6MYO) has no sequence corresponding to the N-terminal extension of Sdh4, which also approaches the Q site in the yeast structure. **b,** Cross-eyed stereo viewing of superimposed Sdh3 and Sdh4 in the vicinity of the inhibitor, based on the four segments in panel a. The backbone is rendered as cartoon with Sdh3 in ocre and Sdh4 in blue for CII-bix; both chains are grey for the avian enzyme. Side chains are ball-and-stick with yellow carbons for CII-bix and grey for avian. Side chains for Sdh3 W194 and corresponding residue in 6MYO are shown, Sdh2:237 is omitted for clarity. Potential H-bonds for CII-bix are shown as white dotted lines. Significant differences discussed in the text are Sdh3 Ser93 (replaces Leu) and Sdh4 Glu112 (replaces Gly) and the involvement of the N-terminal residue of Sdh4. Also shown are the heme with its axial ligand residues in 6MYO, and the corresponding residues in CII-bix making a direct H-bond. The side chain of Sdh3 Leu100 (replaces Gly) fills the space otherwise occupied by the edge of the heme ring in heme-containing SQRs.

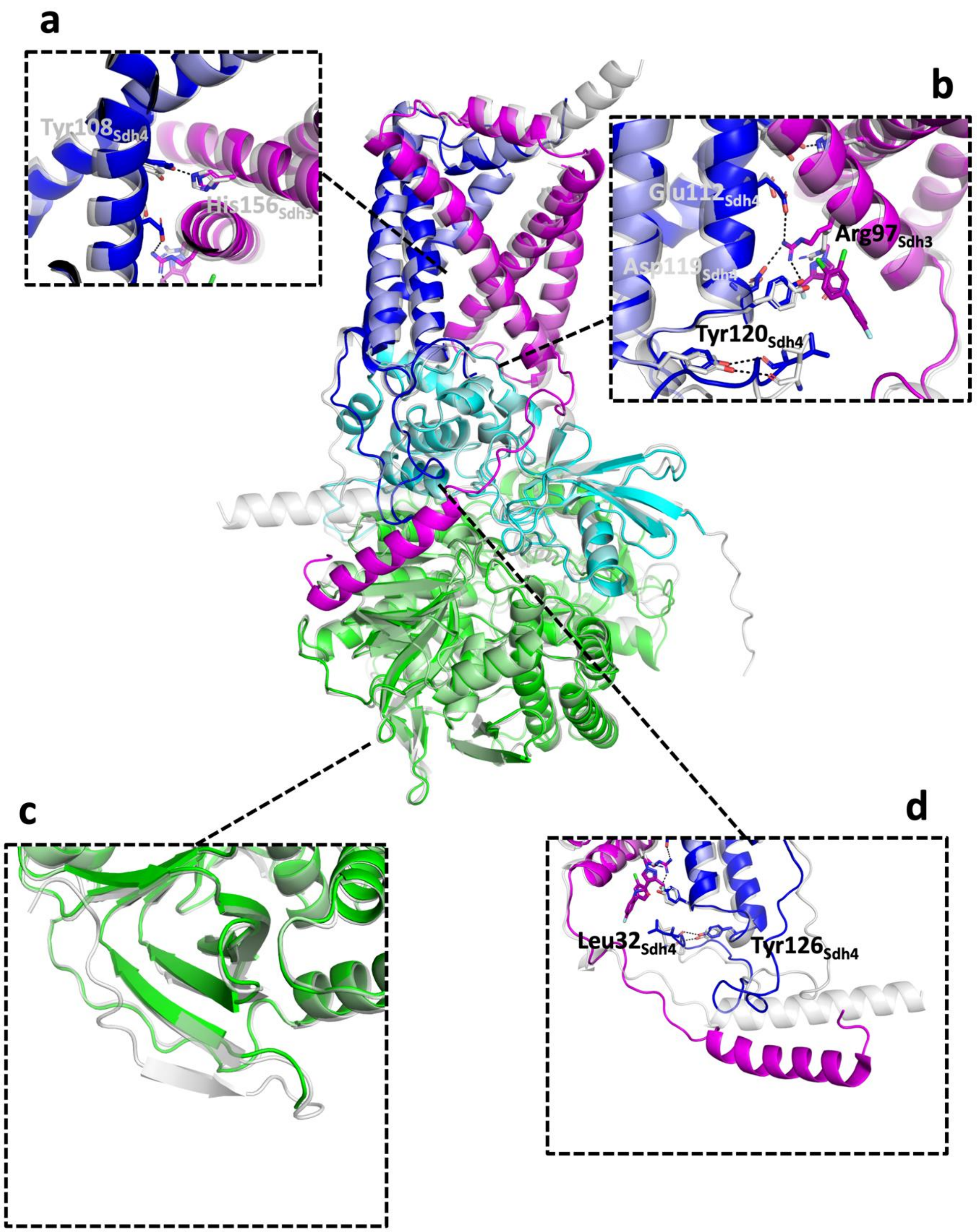

Supplementary Fig. 12: Superimposition of the cryo-EM model (CII-bix) with a complex composed of the AlphaFold models. The cryo-EM model is in the same colors as in Fig. 1, while the AlphaFold models are colored in gray. Specific differences are highlighted in the four panels.

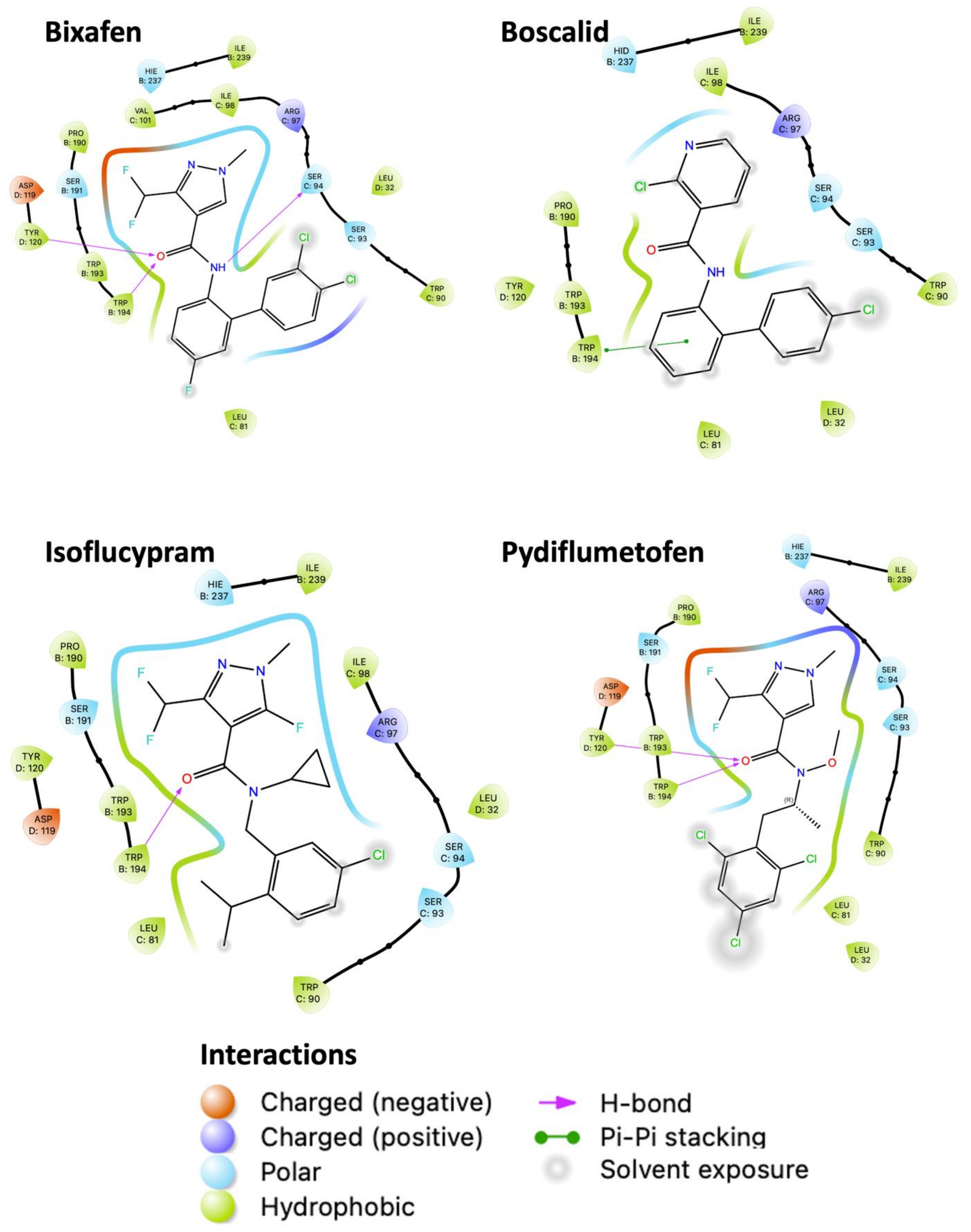

Supplementary Fig. 13: 2D stick diagram of the inhibitors modeled in the active site. Pink arrows represent H-bonds and the green connector bar the π-stacking interaction. Red and blue colors indicate negative and positive charges interactions, light blue polar interactions and green hydrophobic interactions. Blue arrows indicate H-bonds and the red line π-π stacking interaction. The solvent exposed residues are grey shaded.

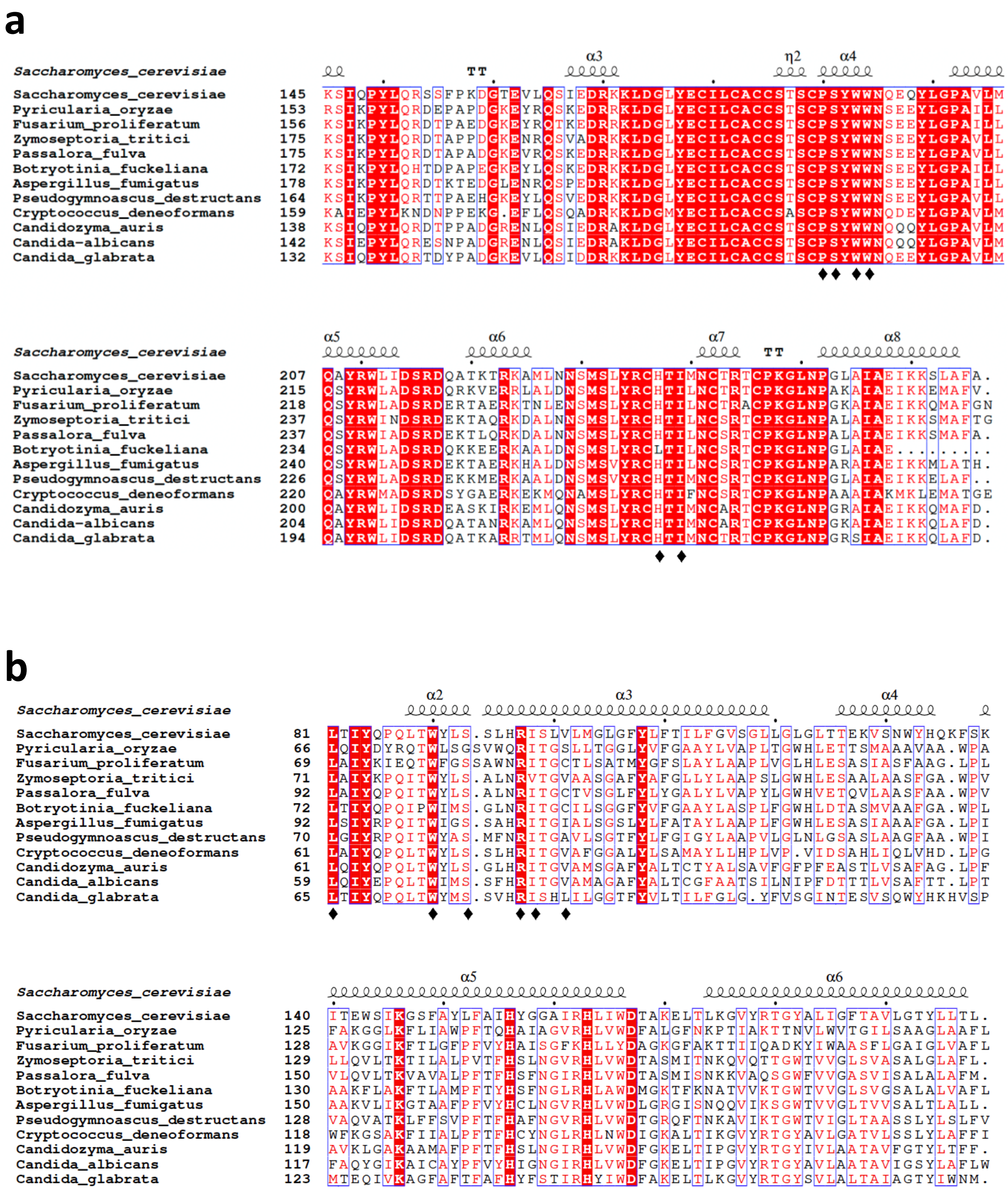

Supplementary Fig. 14: Alignment of Sdh2 and Sdh3 of *S. cerevisiae* and selected pathogenic fungi as per Fig. 7. Residues that interact with bixafen in *S. cerevisiae* are indicated with diamonds.

a

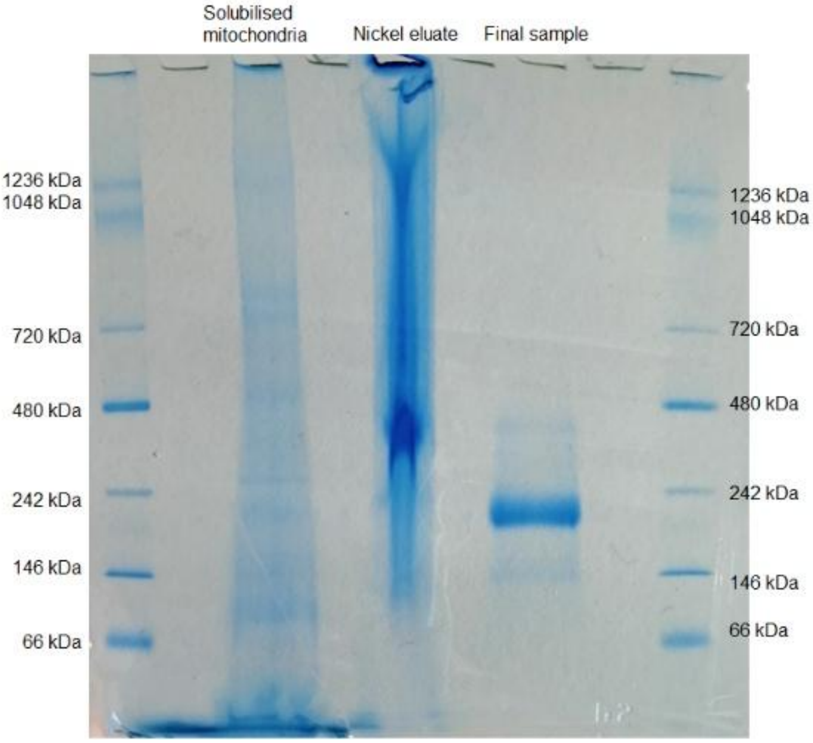

b

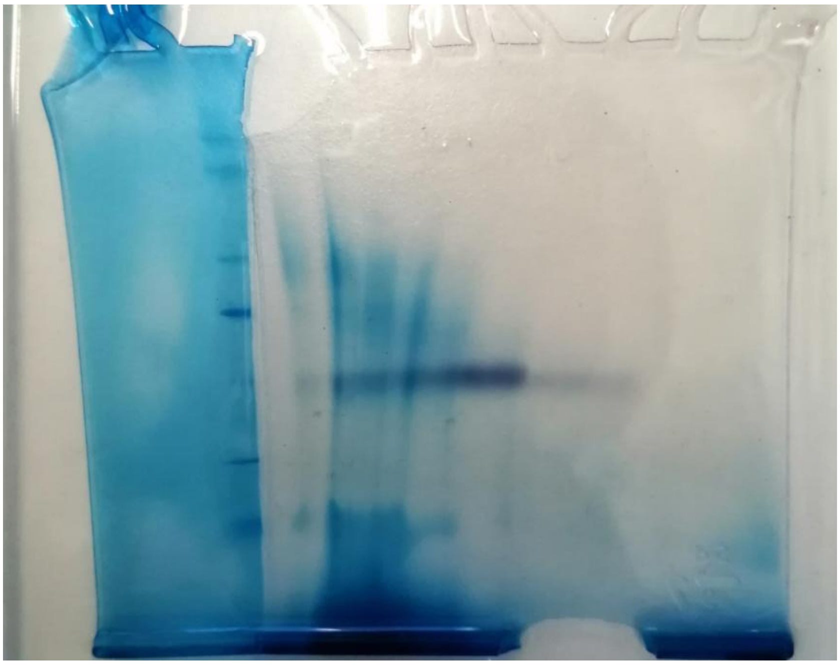

Supplementary Fig. 15: Original uncropped gels presented in Supplementary Fig. 1c and d. a, BN-PAGE gel of different fractions collected on CII purification. The lane labelled ‘Final sample’ is presented in Supplementary Fig. 1c. **b**, CN-PAGE gel cut in 2 segments: the smallest to the left was stained with Coomassie to reveal the molecular weight marker bands; the biggest to the right was incubated with in-gel activity solution to reveal the presence of active CII. The 5th lane represents the ‘Final sample’ used for structure determination presented in Supplementary Fig. 1d.

Supplementary Table 1: Alignment to AlphaFold models.

Chain	Model accession	RMSD, Å*	Residues matched
Sdh1	Q00711	0.488	462/597
Sdh2	P21801	0.417	219/246
Sdh3	P33421	0.417	115/148
Sdh4	P37298	0.629	104/150

*RMSD between AlphaFold models and cryo-EM models

Supplementary Table 2: Docking results with SDHIs.

S/N	Entry Name	Docking score*	Glide Emodel	MW
1	Bixafen	-8.74	-77.34	414.21
2	Boscalid	-9.53	-78.25	343.21
3	Isoflucypram	-6.84	-78.25	399.84
4a+	Pydiflumetofen	-4.26	-57.49	426.67
4b+	Pydiflumetofen	-3.83	-49.65	426.67

*****Glide G score values are exactly the same to the Docking score values

+Pydiflumetofen docking generated two conformations of the trichloro-phenyl group

Supplementary Table 3: Sdh4 homologs of pathogenic fungi of critical interest for disease control in human and animal health and agriculture. The UniProt entry code for each homolog used for sequence alignment is given as well as the level of threat as currently recognized by the World Health Organization (WHO), Agriculture and Horticulture Development Board UK (AHDB), National Institutes of Health USA (NIH) or World Organization of Animal Health (WOAH).

**Organism**	**UniProt Entry Code**	**Level of threat and function/cause**	**Source**
Candida glabrata	Q6FU68	Critical threat, invasive fungal infection in immunosuppressed individuals	WHO
Candida albicans	Q5AJZ8	Critical threat, invasive fungal infection in immunosuppressed individuals	WHO
Candida auris	A0A2H0ZVQ3	Critical threat, invasive fungal infection in immunosuppressed individuals	WHO
Cryptococcus neoformans	A0A854QQK5	Critical threat, invasive fungal infection in immunosuppressed individuals	WHO
Zymoseptoria tritici	F9X9V6	The most significant and damaging foliar disease on winter wheat	AHDB
Passalora fulva	A0A9Q8LGU8	The most destructive mold affecting tomatoes grown in humid conditions	AHDB
Fusarium proliferatum	A0A1L7V4Q7	High threat, invasive fusariosis, affecting immunocompromised patients	WHO
Pyricularia oryzae	A0A4P7NKH2	The most devastating pathogenic fungi that affects a wide range of cereal plants, especially rice	NIH
Aspergillus fumigatus	A0A229X696	Critical threat, invasive fungal infection in immunosuppressed individuals	WHO
Botrytis cinerea	D2IH01	Grey mold in flowers and fruits like grapes, strawberries, mild infection	AHDB
Pseudogymnoascus destructans	A0A177ANA7	White-nose-syndrome, bat killing fungus	WOAH

Supplementary text

Further details of the structure.

Flavoprotein (Sdh1). The flavoprotein of CII contains the covalently bound flavin (FAD) and the succinate binding site. The mature *S. cerevisiae* protein consists of residues 29 – 640 of the open reading frame (ORF), although there is no experimental evidence of the transit peptide length. Of these the models include residues 44-635, of which 91 in the cap domain have not been modeled (see below), for a total of 518 residues. As expected from sequence homology, the structures of the yeast flavoprotein and iron-protein are very similar to those determined from vertebrate complexes. Excluding six areas with differences described below, the remaining 444 residues align with the avian (PDB ID 6MYO) or human (PDB ID 8GS8) homologs with RMSD 0.69 and 0.62 Å (CII-bix) or 0.79 and 0.69 Å (CII-nat); for the main-chain atoms.

Comparing with the vertebrate structures, the largest area of difference is the so-called cap domain, residues 285-399 in the yeast protein. This domain is present in all succinate:quinone oxidoreductase superfamily flavoproteins and is known to be mobile, having different positions in different structures of the same protein. In the structures here the cap domain is poorly ordered, and two stretches totaling 91 residues could not be unambiguously modeled (residues 290-330 and 340-390). The two strands by which it is connected to the main (FAD) domain diverge from those in the structures with closed cap with a hinge around residues 285 and 397. Aligning the most reliable parts of the cap domain (outgoing strand, helix at 331-343, and returning strand) with the closed cap of the avian structure (6MYO) requires an additional ~19° rotation compared to the rest of the flavoprotein, suggesting the cap is partially open. Comparison of succinate:quinone reductase (SQR) flavoproteins

with open cap domain to ones with closed cap domains (PDB IDs 2WDQ and 6MYO) showed rigid motion of the cap with a hinge near the same place, and angles of about 20° (PDB ID 6VAX), 44° (PDB ID 6C12) or 39° (PDB ID 8DYD).

Otherwise, the differences are small and local: the first two residues of the model (leading into the disordered N-terminus), residues A415 to A421 (a surface loop between two beta strands, in which the vertebrate proteins have a deletion of two residues compared to yeast), a stretch of five surface residues (472-478) that are significantly displaced from their position in the vertebrate complex, three residues (596 - 598) at the junction between the Helical and C-terminal domain (replaced in vertebrates by an external loop due to an insertion of 17 residues), residues 607 to 611 (a turn between strands of the beta hairpin in the C-terminal domain, positioned differently than the vertebrate counterparts even though the number of residues is the same), and the C-terminus after residue 623 (the yeast structure is diverging from the vertebrates and becoming disordered). The final 5 residues could not be modelled in either yeast structure.

Disorder of the C-terminus of the flavoprotein may result from an open cap domain. In vertebrate and bacterial SQR structures in which the cap domain is closed, both the cap and the C-terminus are well ordered. The terminal tyrosine residue, and the arginine two residues before that (corresponding to Tyr640 and Arg638 in yeast) form ion pairs or H-bonds with residues in the cap domain. It has been proposed that these bonds have to break for the cap to open. Loss of these interactions may result in the C-terminus becoming mobile and disordered.

There is a metal-binding site adjacent to the dicarboxylate site with roughly octahedral ligation from five backbone carbonyl oxygens and the hydroxyl oxygen of Tyr399. The distances measured between the metal and the carbonyl oxygens (2.71 Å for Glu433, 2.79 Å for Gly402, 2.95 Å for Ala435, 3.07 Å for Asn400 and 3.46 Å for

Met401) are consistent with the presence of a potassium ion for which longer distances are expected compared to e.g. sodium, magnesium or calcium[1]. In the vicinity of the dicarboxylate and metal-binding site we also observe a rare non-proline cis peptide bond between Val437-Ser438. These two features are conserved in the flavoproteins of vertebrate, *Ascaris* and *E. coli* SQR, and the metal site is also present in the quinol:fumarate reductases (QFR) from *E. coli* or *Wollinella succinogenes* and even the soluble flavocytochrome fumarate reductase of *Shewanella* sp.

Iron-sulfur protein (Sdh2). The mature protein comprises residues 21 to 266, of which the models are complete to the C-terminus but nine (CII-nat), or ten (CII-bix), N-terminal residues are either disordered and not modelled or they belong to the transit peptide (for UNIPROT entry P21801 precise cleavage position has not been experimentally determined).

The iron-sulfur protein is made up of two domains, an N-terminal domain resembling plant ferredoxins with one Fe_2S_2 cluster, and a C-terminal domain resembling bacterial ferredoxins with one Fe_4S_4 and one Fe_3S_4 cluster. In the yeast structure the dividing point is around residue 137. For the CII-nat structure, the N-terminal domain aligns with the avian structure with RMSD 0.36 Å, excluding a segment 111 - 117 which is slightly displaced relative to its position in the vertebrate structures. The second domain aligns with RMSD 0.51 Å excluding an external loop at 154-162 which has one more residue in vertebrates and folds differently. Aligning both domains simultaneously with the same exclusions gives an RMSD of 0.57 Å. The corresponding values for the CII-bix structure are 0.37 Å for the first domain, 0.47 for the second, and 0.54 Å for the whole. The relative orientation between the two domains differs from that in vertebrates by only 3.6° (CII-nat) or 3.5° (CII-bix), which

may not be significant. Overall, the structure is essentially the same as the corresponding protein in vertebrate CII.

Small subunits. The membrane anchor of CII is a heterodimer of two small subunits (Sdh3 and Sdh4) with similar fold. Each subunit has three transmembrane helices (TMHs, Supplementary Fig. 7). The first two TMHs from each subunit come together to form a four-helix bundle that normally encloses the heme B, coordinated by histidine residues in TMH2 of each subunit. The N-termini of each are on the proximal (matrix) side of the membrane. Variable features include long extensions on the N-terminus before TMH1, that interact with the extrinsic subunits Sdh1 and Sdh2, and amphipathic "in-plane" helices on the other (IMS) side of the membrane. In mitochondrial CII including yeast, Sdh3 has an in-plane helix (α3) between TMH1 and TMH2, while Sdh4 has one (α4’) at the C-terminus after TMH3 (Supplementary Fig. 7a).

The small subunits of yeast are structurally similar to their vertebrate counterparts in regions involved in the quinone reduction site, interactions with Sdh2, and heme binding residues (or the Tyr:His pair that replace them in yeast). Specifically, residues C84-104 (the proximal end of TMH1 (α2) and 4 residues before), C156-188 (proximal ends of TMH2 and TMH3, and the linker between them) in Sdh3 and D:108-120 (proximal end of TMH2) of Sdh4 can be superimposed with the avian counterpart (PDB ID 6MYO) with RMSD 0.62 Å for main-chain atoms.

Larger structural differences, as well as residue substitutions that contribute to holding the subunits together in the absence of heme, have been discussed in the main article.

Comparison with the AlphaFold models. Amongst the critical stabilizing interactions that cannot be predicted by AlphaFold are the absence of the $His156_{Sdh3}$-$Tyr108_{Sdh4}$ H-bond (Supplementary Fig. 12a) and the $Glu112_{Sdh4}$-$Arg97_{Sdh3}$-$Asp119_{Sdh4}$-$Tyr120_{Sdh4}$ H-bond network (Supplementary Fig. 12b).

Other unique elements of the yeast structure revealed by cryo-EM include the different position of the Sdh3 N-terminal α-helix, which in the cryo-EM structures interacts with Sdh1 (Supplementary Fig. 12 main panel). The Sdh2 AlphaFold model contains 10 additional residues at the N-terminus, however the presence of these in the mature complex has not been experimentally demonstrated in the curated UniProt entry Q08230 (Supplementary Fig. 12 main panel). The Sdh4 cryo-EM models have a shorter C-terminal α-helix, which can be due to an increased flexibility arising from the sequence extension and poly-histidine tag addition for ease of purification (Supplementary Fig. 12 main panel). The N-terminus of Sdh1 contains one additional β-strand as part of the first β-sheet in the AlphaFold model, which is absent in our cryo-EM model or previously determined crystal structures of other forms of the enzyme (Supplementary Fig. 12c).

Finally, the N-terminal coil of Sdh4 shows some conservation between our cryo-EM structures and the predicted AlphaFold model, most likely because of the recognized H-bond between $Tyr126_{Sdh4}$ and $Leu32_{Sdh4}$ (Supplementary Fig. 12d).

Supplementary Reference

1 Harding, M. M. Small revisions to predicted distances around metal sites in proteins. *Acta Crystallogr D Biol Crystallogr* **62**, 678-682 (2006). https://doi.org/10.1107/S0907444906014594
