# Supplementary material for: Cryo-EM structure of bixafen-bound S. cerevisiae complex II unravels SDHI specificity against pathogenic fungi
Source: Commun Biol. 2026 Jan 28;9:517. doi: 10.1038/s42003-026-09617-8 (PMC13066397; doi:10.1038/s42003-026-09617-8)
Supplement: Supplementary file 3 — Description of Additional Supplementary Files [file 42003_2026_9617_MOESM3_ESM.docx]

**Description of Additional Supplementary File**

File name: Supplementary Data

Description: The source data behind the graphs in Supp Fig 1
